# Supplementary figures and images for: Bud23 promotes the final disassembly of the small subunit Processome in Saccharomyces cerevisiae
Source: PLoS Genet. 2020 Dec 11;16(12):e1009215. doi: 10.1371/journal.pgen.1009215 (PMC7758049; doi:10.1371/journal.pgen.1009215)

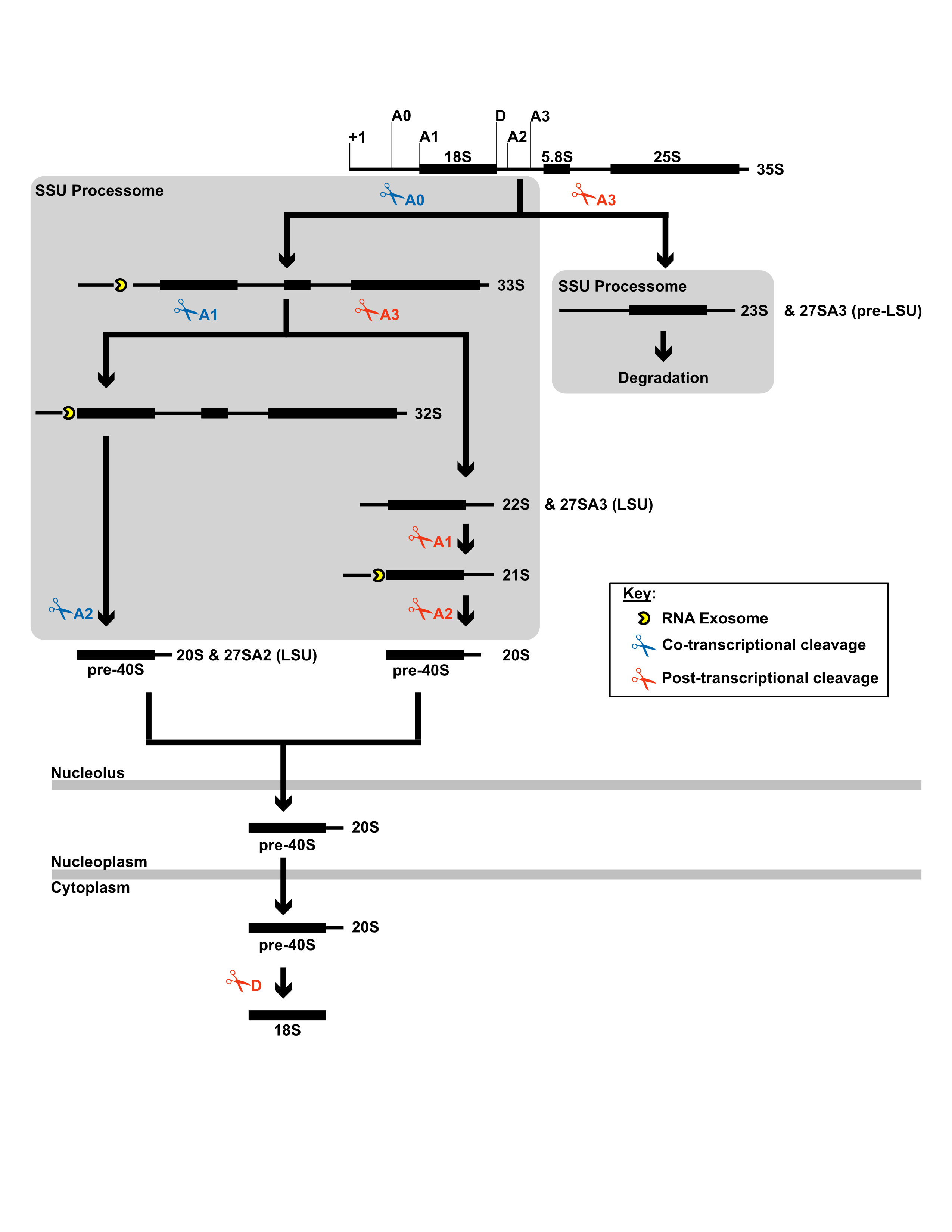

Supplement: S1 Fig — Endonucleolytic processing of the pre-18S rRNA at sites A0, A1, and A2 (or A3) occurs within the context of the SSU Processome. Events occurring co- or post-transcriptionally are denoted by blue and red scissors, respectively. Cleavage at either the A2 or A3 sites liberates the SSU precursors from the LSU precursors containing the 27SA2 and 27SA3 rRNA intermediates, respectively. In rapidly dividing cells processing of A0, A1, and A2 appear to occur co-transcriptionally in a sequential order to produce the 20S rRNA intermediate. The RNA Exosome exonucleolytically degrades the A0- and A1-cleaved 5’ ETS fragments. When A1 or A2 cleavage is delayed, post-transcriptional cleavage at A3 occurs to produce the 22S or 21S rRNA, and A1 and A2 cleavage instead occur post-transcriptionally to generate the 20S rRNA. When SSU Processome function is entirely precluded, cleavage at A3 produces the 23S rRNA that becomes degraded. The 20S rRNA is a component of pre-40S intermediates that are exported to the cytoplasm where an endonuclease processes the D site to yield the 18S rRNA. (TIFF) [file pgen.1009215.s001.tiff]

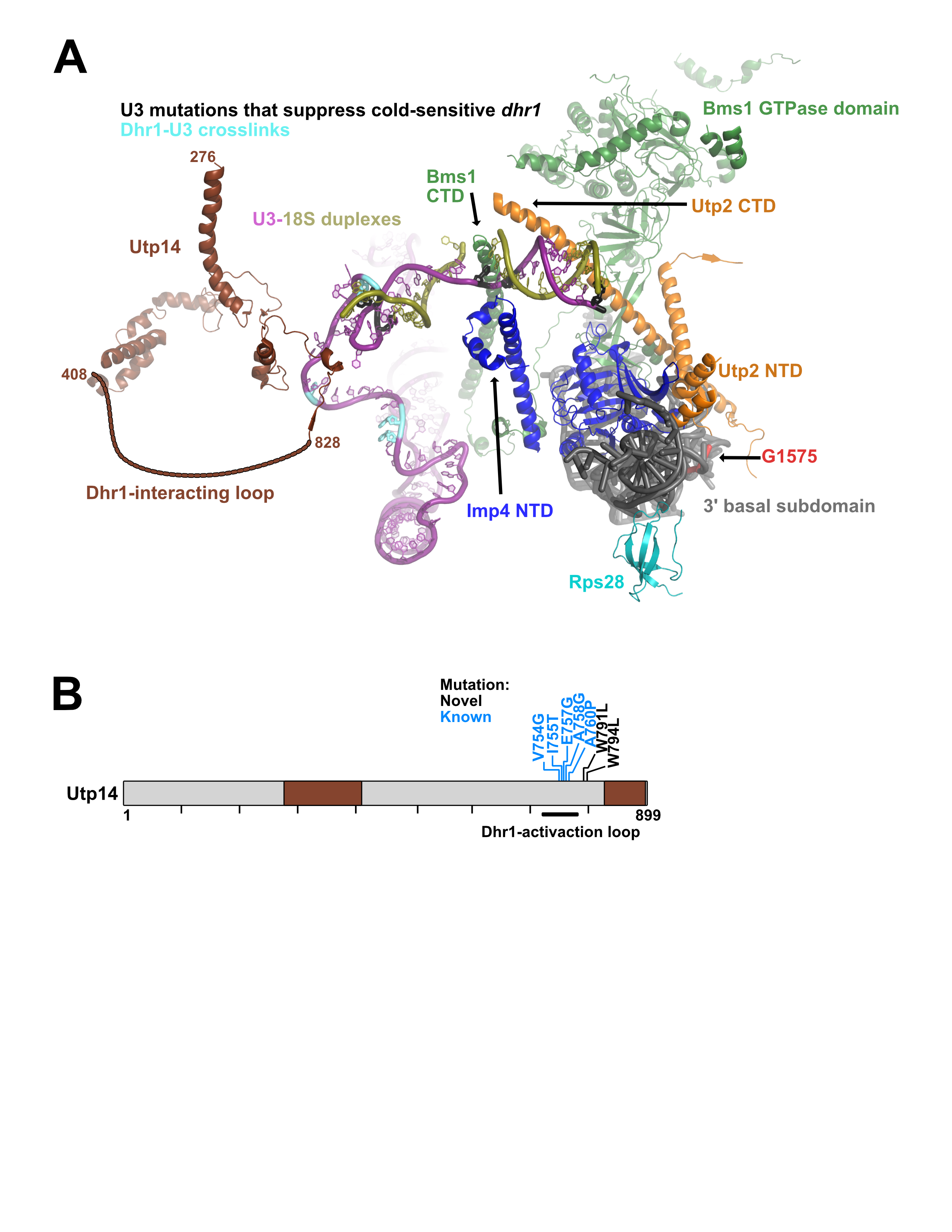

Supplement: S3 Fig — (A) The location of the resolved segments of Utp14 (brown) in the SSU Processome. A contour line indicates the unresolved region of Utp14 where the Dhr1-interaction surface and bud23Δ-suppressing mutations are located. The U3 snoRNA binding site of Dhr1 and U3 mutations that suppress a cold-sensitive Dhr1 mutant [29] are indicated by cyan and black sticks, respectively. Bms1, Imp4, Rps28, Utp2, and the 3’ basal subdomain RNA are shown for reference. (B) A cartoon of Utp14 primary structure indicating the position of its resolved portions and the bud23Δ-suppressing mutations reported here (black) and previously (light blue) within its Dhr1-activaction loop [30]. (TIFF) [file pgen.1009215.s003.tiff]

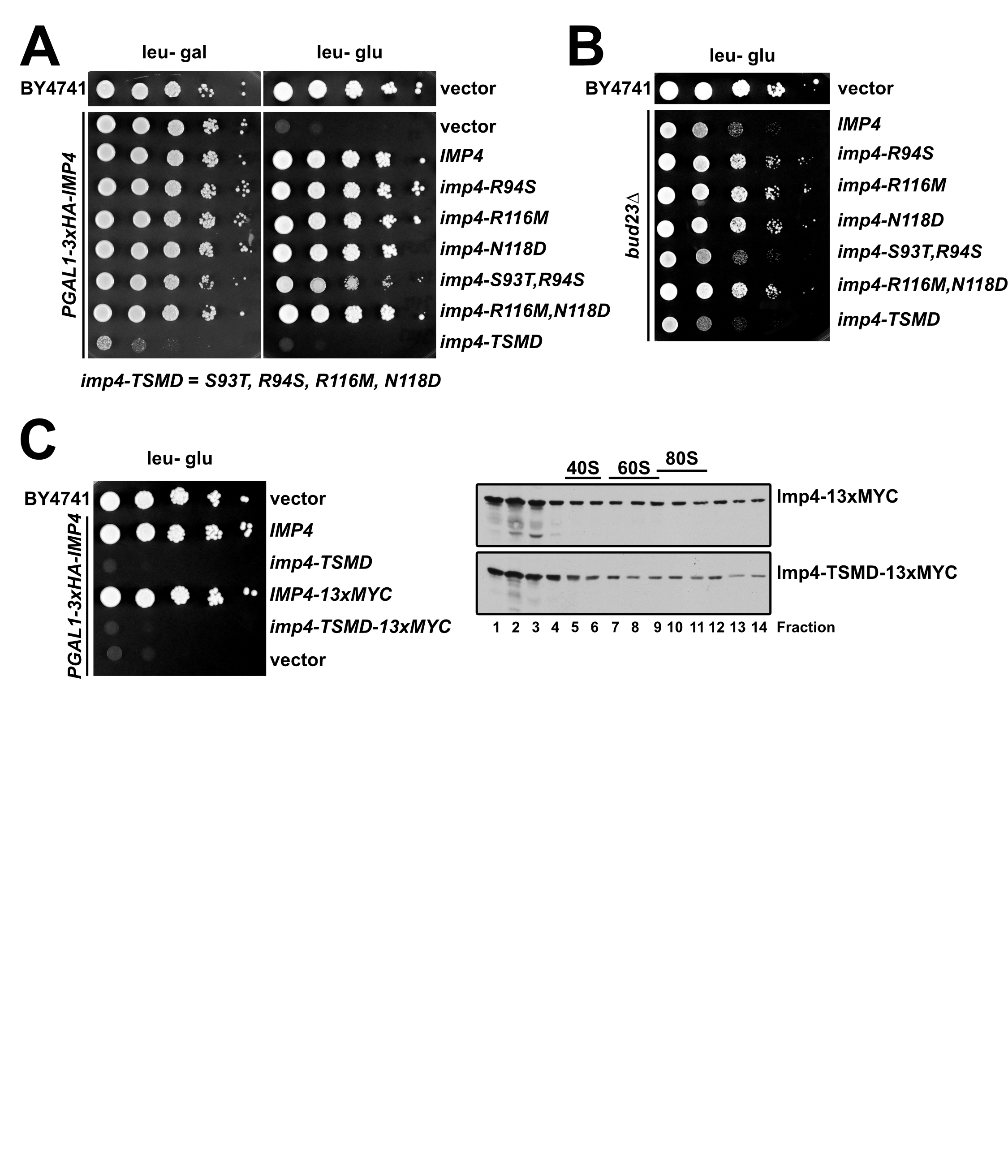

Supplement: S4 Fig — (A) Complementation by the indicated IMP4 alleles as shown by 10-fold serial dilutions of wild-type cells (BY4741) or PGAL1-3xHA-IMP4 (AJY3822) cells harboring either an empty vector (pRS315) or vectors encoding the indicated alleles of IMP4 spotted on SD-Leu media containing galactose or glucose and grown for two days at 30°C. (B) Suppression of the growth defect of bud23Δ by the ectopic expression of the indicated IMP4 alleles as shown by 10-fold serial dilutions of wild-type cells (BY4741) or bud23Δ (AJY2676) cells harboring either an empty vector (pRS315) or vectors encoding the indicated alleles of IMP4 spotted on SD-Leu- media containing glucose and grown for two days at 30°C. (C) Left panel: Complementation of Imp4-13xmyc as shown by 10-fold serial dilutions of BY4741 cells or PGAL1-3xHA-IMP4 (AJY3822) cells harboring either an empty vector (pRS315) or vectors expressing tagged or untagged versions of the indicated IMP4 alleles spotted on SD-Leu- media containing glucose and grown for two days at 30°C. Right panel: The sucrose density gradient sedimentation of ectopically expressed Imp4-13xMYC and Imp4-TSMD-13xMYC. Extracts were prepared from BY4741 cells harboring vectors expressing Imp4-13xMYC (pAJ2720) or Imp4-TSMD-13xMYC (pAJ2723) as described in the Materials and methods of the main text except 150 μg/ml CHX was used. For each sample, 9 A260 units of clarified extract was separated on sucrose density gradients prior to fractionation. Proteins from each fraction were precipitated with TCA and subjected to Western blot analysis using anti-c-myc antibody (Covance). (TIFF) [file pgen.1009215.s004.tiff]

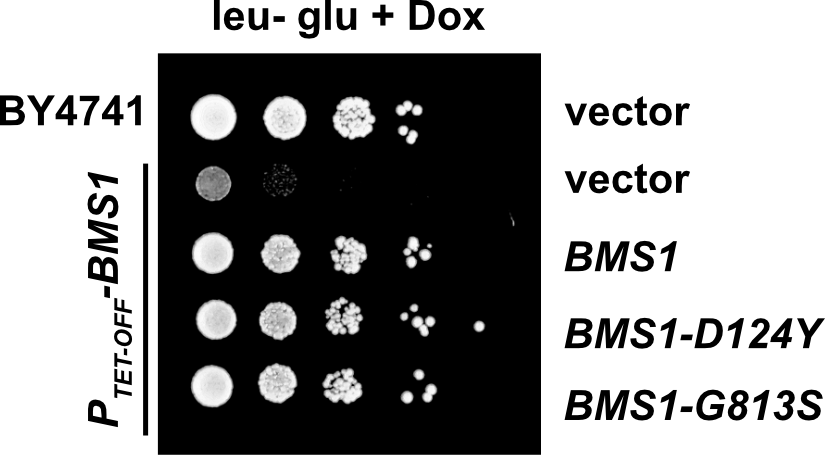

Supplement: S5 Fig — Complementation of selected BMS1 alleles in BUD23-replete cells as shown by 10-fold serial dilutions of BY4741 cells and PTETOFF-BMS1 (AJY4377) cells harboring either an empty vector (pRS415) or vectors encoding the indicated BMS1 alleles spotted on SD-Leu- media containing glucose and 20 μg/mL doxycycline and grown for three days at 30°C. (TIFF) [file pgen.1009215.s005.tiff]

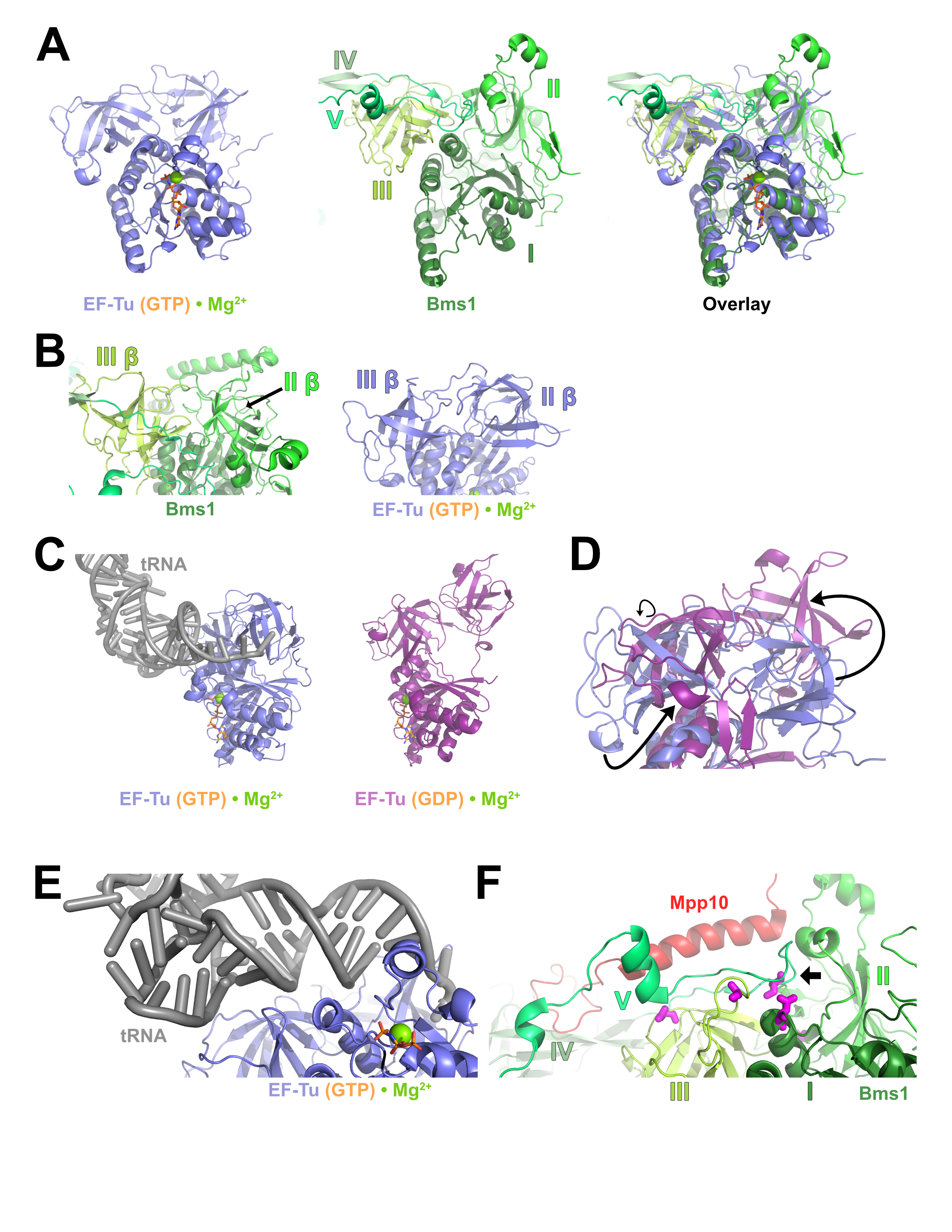

Supplement: S6 Fig — (A) Structural alignment of EF-Tu bound to the non-hydrolysable GTP analog, GDPNP (slate blue, PDB 1B23) to domain I of Bms1 (from PDB 5WLC) is shown. Bms1 is colored by domains as in Fig 4D; the GTP analog and magnesium ion bound to EF-Tu are shown as orange sticks and green sphere. Structures are shown individually (left, middle) and as an overlay (right). (B) A view of domains II and III of Bms1 compared to those of EF-Tu shows that the two domains adopt beta barrels in similar conformations. (C) GDPNP-bound EF-Tu forms a complex with tRNA, while GDP-bound EF-Tu (deep purple, PDB 1EFC) does not. (D) Conformational differences in the beta-barrel domains of GDP and GTP-bound EF-Tu suggest that these domains rotate away from one another upon GTP hydrolysis to promote tRNA release. (E) The amino-acyl tRNA contacts GDPNP-bound EF-Tu through its two beta barrel domains. (F) Bms1 in the same orientation as EF-Tu in panel E. The unstructured loop of domain V that connects it to domain IV (denoted by the black arrow) and an N-terminal helix of Mpp10 (red) contacts domains II and III of Bms1 in a manner reminiscent of how tRNA interacts with GDPNP-bound EF-Tu. The mutated residues that suppress bud23Δ are shown as magenta sticks. (TIFF) [file pgen.1009215.s006.tiff]

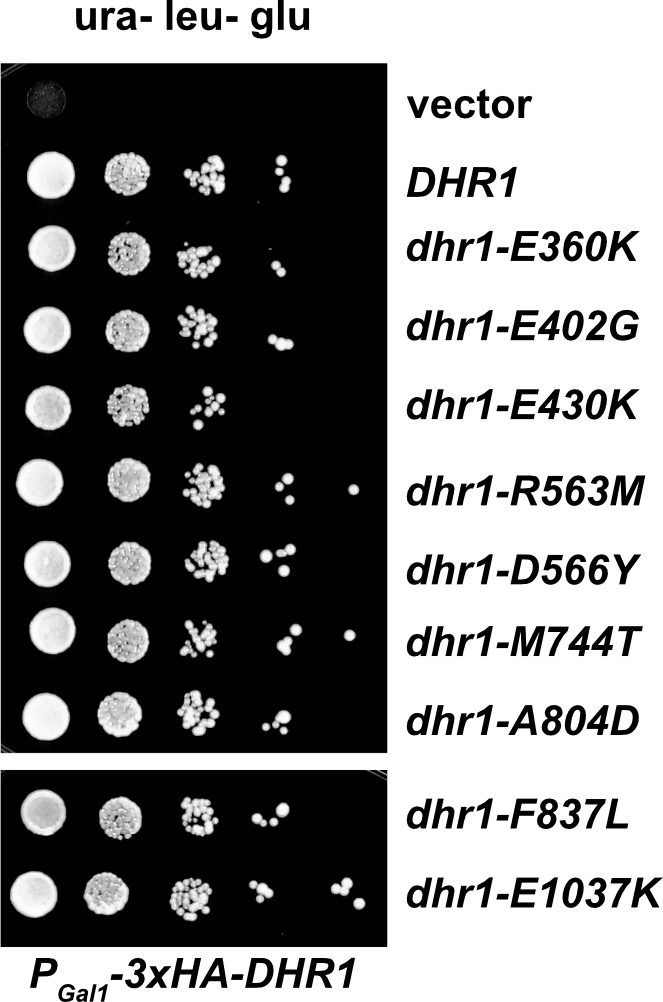

Supplement: S7 Fig — Complementation of select DHR1 alleles in BUD23-replete cells as shown by 10-fold serial dilutions of PGAL1-3xHA-DHR1, PGAL1-3xHA-UTP14 (AJY4605) cells harboring a vector expressing UTP14 (pAJ1919; [45]) and either an empty vector (pRS415) or vectors encoding the indicated DHR1 alleles spotted on SD-Leu-Ura- media containing glucose and grown for two days at 30°C. (TIFF) [file pgen.1009215.s007.tiff]

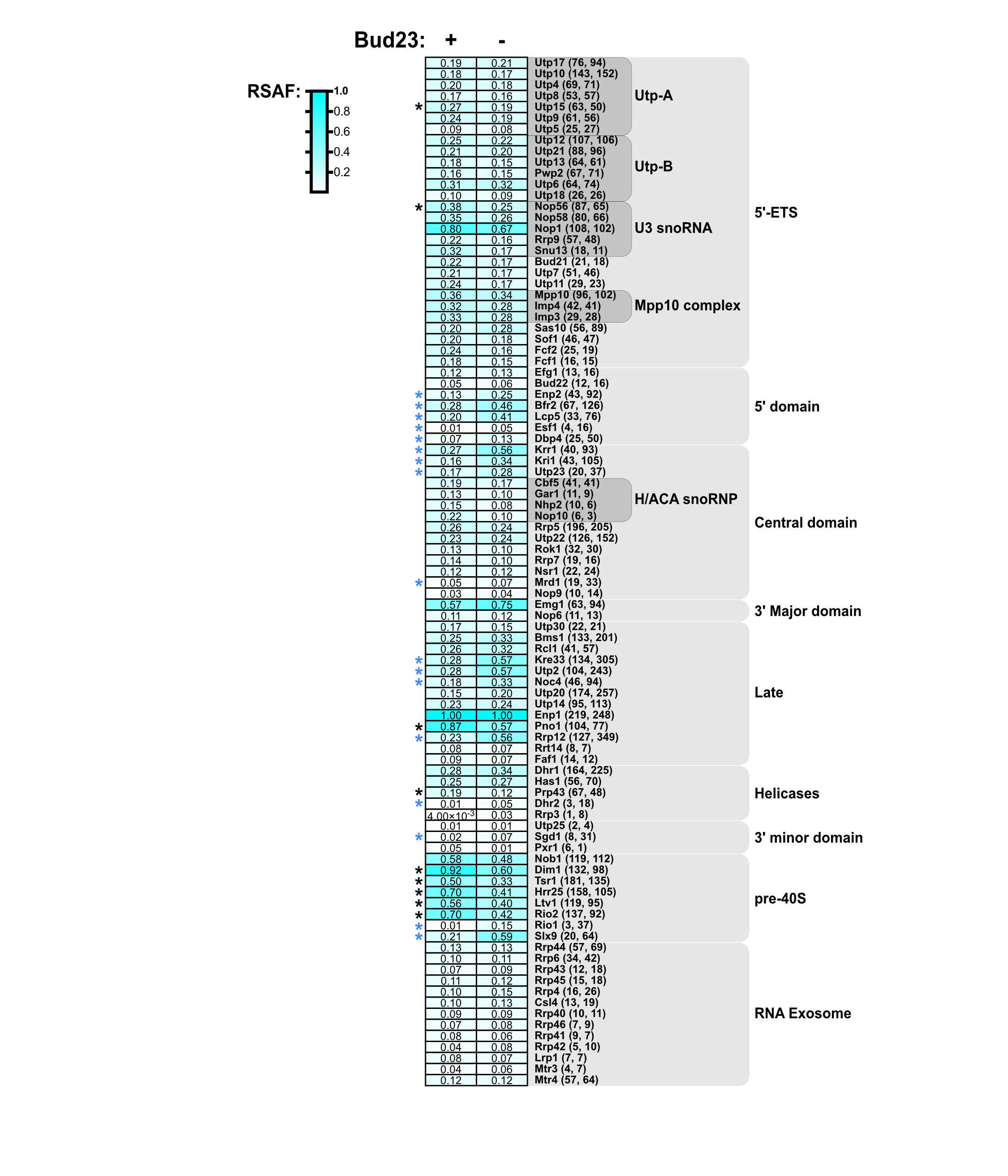

Supplement: S8 Fig — Related to Fig 7. A heatmap of SSU biogenesis proteins that co-immunoprecipitated with Enp1-TAP in the presence (+) or absence (-) of Bud23 is shown. The scale spanning from 0 (white) to 1 (cyan) reflects the relative spectral abundance factor (RSAF). The RSAF was calculated by first normalizing the total number of spectral counts identified for a given protein to its molecular weight; these values were further normalized to the bait, Enp1, to reflect stoichiometry. RSAF values for each protein are shown within each cell. For each protein, the number of spectral counts identified in the presence or absence of Bud23 are shown in parentheses, respectively. Proteins that showed a significant increase or decrease relative to the + Bud23 sample and are listed in S9 Fig are denoted by an asterisks (*) colored blue or black, respectively. Proteins are grouped according to [7] or by known function. Heatmaps were generated in Graphpad Prism version 8.3.0 (328) for Mac iOS. The complete data for this figure are available in S2 Table. (TIFF) [file pgen.1009215.s008.tiff]

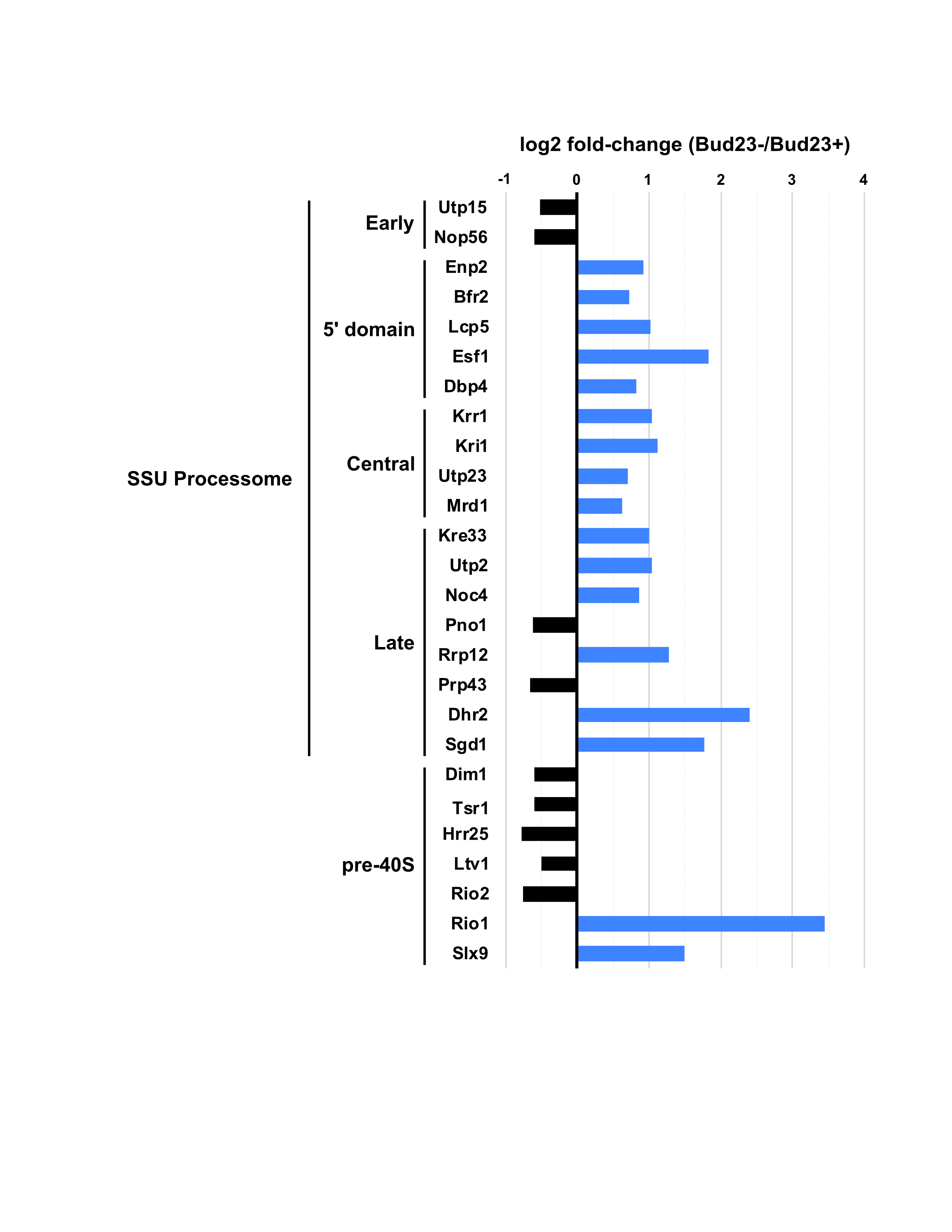

Supplement: S9 Fig — Related to Fig 7 and S8 Fig. Mass spectrometry analysis of total proteins that co-precipitated with Enp1. Proteins that showed a significant log2 fold-change difference in the absence or presence or Bud23 are shown. Total number of peptides identified for each protein was normalized to molecular weight then further normalized to the bait to generate RSAF values (see Materials and methods) which were used to calculate the log2 fold-change between the mutant and wild-type samples. Proteins displaying a ± 0.5-fold change or more with a difference of greater than 10 total spectral counts are plotted. Proteins are grouped according to when they first bind to pre-ribosomes [7]. The complete mass spectrometry data are available in S2 Table. (TIFF) [file pgen.1009215.s009.tiff]

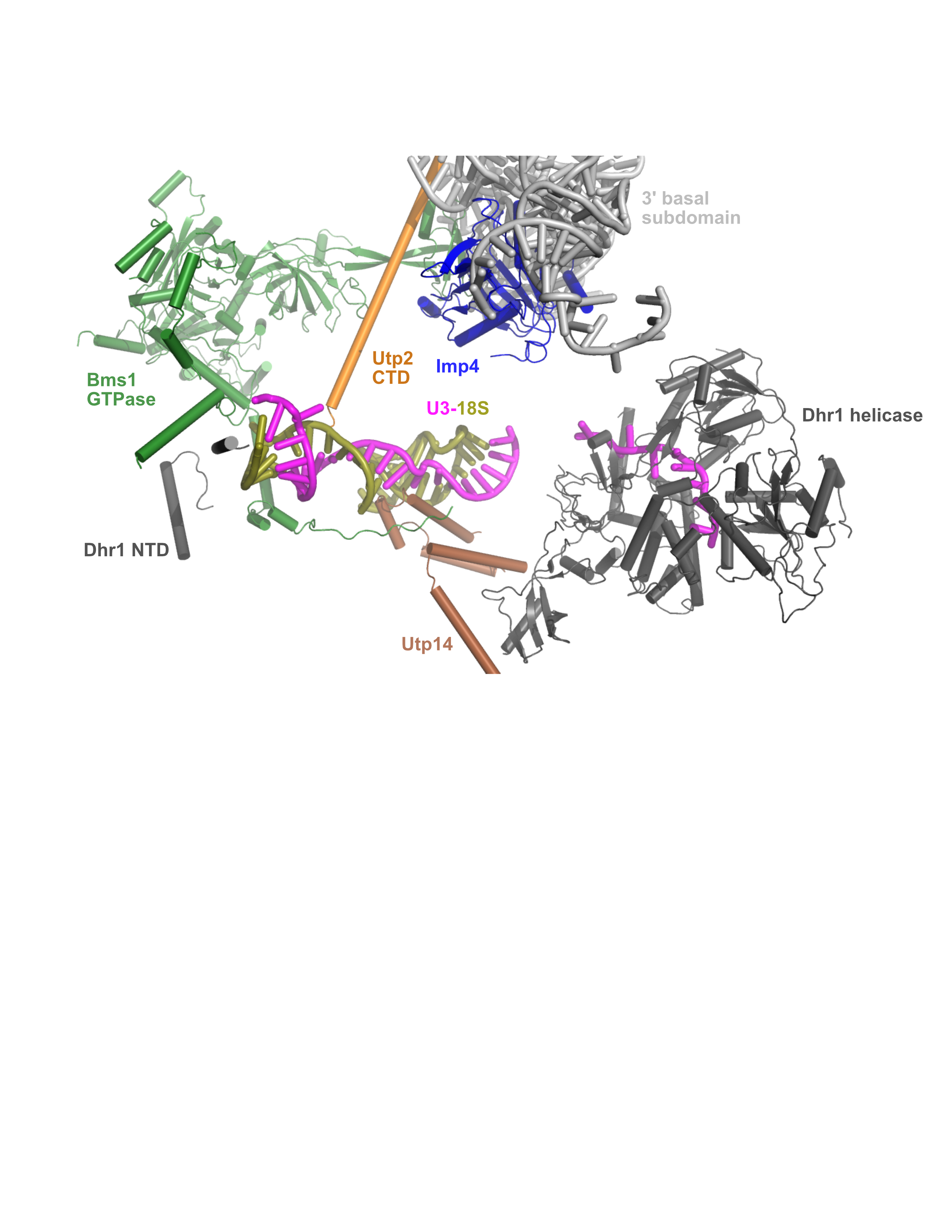

Supplement: S10 Fig — The GTPase core of Bms1 (green) and the helicase core of Dhr1 (dark gray) are opposite one another in the Dis-C complex (PDB 6ZQG), but part of the N-terminal domain (NTD) of Dhr1 interacts with Bms1 on the distal side of the U3-18S heteroduplexes (magenta/gold) that Dhr1 unwinds. The 3’ basal subdomain (light gray), Imp4 (blue), Utp2 C-terminal domain (CTD; orange), and Utp14 (brown) are shown for reference. (TIFF) [file pgen.1009215.s010.tiff]
