# Supplementary material for: Bud23 promotes the final disassembly of the small subunit Processome in Saccharomyces cerevisiae
Source: PLoS Genet. 2020 Dec 11;16(12):e1009215. doi: 10.1371/journal.pgen.1009215 (PMC7758049; doi:10.1371/journal.pgen.1009215)
Supplement: Attachment — Submitted filename: Response to Reviewers.pdf [file pgen.1009215.s013.pdf]

Reviews and responses from eLife submission

Dear Dr Johnson,

Thank you for submitting your work entitled "Bud23 promotes the progression of the Small Subunit Processome to the pre-40S ribosome in Saccharomyces cerevisiae" for consideration by eLife. Your article has been reviewed by 3 peer reviewers, and the evaluation has been overseen by a Reviewing Editor and a Senior Editor. The following individuals involved in review of your submission have agreed to reveal their identity: Jonathan P Staley (Reviewer #1); Katrin Karbstein (Reviewer #2); David Tollervey (Reviewer #3).

Our decision has been reached after consultation between the reviewers. Based on these discussions and the individual reviews below, we regret to inform you that your work will not be considered further for publication in eLife.

Please accept our apologies for the delayed review resulting from the pressure COVID-19 has imposed on the process. All three reviewer agreed that the genetics and suppressor analyses are rigorous, informative, and quite satisfying in the context of the structure. All three reviews also felt, however, that a solid conclusion about Bud23 function cannot be made from the data or easily with follow up work. Additionally, two reviewers felt the model did not easily make sense with respect to Dhr1. Nevertheless, all three reviewers felt the work was appropriate for immediate publication in a more specialized journal.

* Portable peer review *
We would be happy to transfer the peer reviews, along with the reviewers' names (where they agree), to another journal of your choice. For example, in the last 12 months, we have facilitated the transfer of review material to a range of journals, including Biology Open, BMC Biology, EMBO Reports, eNeuro, the PLOS journals, the Journal of Cell Biology, Journal of Neuroscience, and the Journal of General Physiology. Such transfers are only undertaken in response to an explicit request by the corresponding author. For neuroscience submissions, please note that we participate within the Neuroscience Peer Review Consortium (http://nprc.incf.org/index.php/about/information-for-authors/).

Although the sharing of peer reviews can potentially help to save time, the editors of the receiving journal may decide to consult new referees. In addition, not all reviewers provide us with permission to share their identity with another journal. If you would like the eLife reviews to be taken into account during submission to another journal, please submit your article directly using their online manuscript tracking system.

eLife is highly selective, which means that the majority of submissions are rejected, but we thank you for sending your work for review and we hope you will submit to eLife again in the future.

Best wishes,

Jonathan Staley
Reviewing Editor, eLife

James Manley
Senior Editor, eLife

We thank the reviewers for their careful reviews of our manuscript. The major weakness of the previous draft appeared to be how we interpreted the Dhr1 and Utp14 suppressors of *bud23*Δ and how they fit into our model for the role of Bud23 in the disassembly of the SSU Processome. We now realize that we did not fully describe the previous work that guided our thinking in the first submission. In this revision, we have provided a more complete model for how we believe Dhr1 and Utp14 suppressors act and clarified our model for Bud23 function. We feel that this revised manuscript is more compelling and hope that is acceptable for publication in *PLOS Genetics*.

Reviewer #1 (General assessment and major comments (Required)):

In this manuscript, the authors investigate a step in ribosome biogenesis in which the SSU processome transitions to pre-40S ribosome, a key step that involves the release of U3 snoRNA, which otherwise precludes central rRNA folding events. The authors begin with a genetic approach to identify bypass suppressors of a bud23 deletion, a screen that yielded an abundance of suppressors whose interpretation is facilitated by the structure of the SSU processome; strikingly, the suppressors concentrate in the 3' subdomain of 18S rRNA. By northern blot and polysome profiles, the authors verify that the suppressors overcome the bud23 defect in processing, and by two-hybrid the authors satisfyingly reveal that many of the mutations disrupt protein-protein interactions. These parts of the manuscript are thorough and of high scientific standard. However, the remainder of the manuscript, including the model, embodies two contradictions, and the manuscript falls short of a mechanistic understanding of Bud23 function and how it relates to other activities in ribosome biogenesis in a manner that is not easily addressed by follow-up experiments. As such, the manuscript is better suited to a specialty journal.

Major comments:

The authors suggest that binding of Bud23 induces the progression of the SSU processome by releasing factors, because many of the bypass suppressors mutations are expected to or shown to weaken either protein-protein or protein-RNA interactions. In particular, the authors propose that Bud23 enables the activity of Dhr1, an RNA helicase that the authors have shown previously promotes SSU processosome progression by unwinding U3:18S duplexes. A significant challenge to this model, however, is the authors' finding that mutations in both Dhr1 and its activator Utp14 also suppress the bud23 deletion and that the mutations weaken this interaction, suggesting that suppression occurs by slowing Dhr1 the dissociation of U3 - not accelerating it. Indeed, the authors do not consider or test a quite different but viable model: in the bud23 mutation, certain steps occur prematurely (i.e., before Dhr1 activity), and the suppressors act by slowing Dhr1 to "re-order" the events; indeed, out-of-order events do appear to occur in the absence of bud23 - pre-

40S factors Riox1 Slx9 appear to bind prematurely. Importantly, mutations in other RNA helicases have been shown to suppress in this way, by slowing their activity to re-ordering otherwise out-of-order events. If this alternative model were correct, Bud23 would function in an entirely different way than is proposed and in a way that is not illuminated by this manuscript. A useful next step would be to test whether hypomorphic mutations known to compromise the catatlytic activity of Dhr1 also suppress the bud23 deletion, but a resolution of the suppression mechanism or Bud23 function requires significant further study.

We appreciate Dr. Staley's thoughts about the mechanism of suppression of bud23Δ by mutations in Dhr1. Indeed, we have previously proposed such a model (Zhu et al, MCB, 2016), based on the genetic relationship between splicing helicases and poor splicing substrates. In the manuscript we submitted, we failed to adequately address how we envision the Dhr1 mutations act to bypass bud23Δ. We have significantly revised the Discussion to separate the mechanism of suppression by factors at the 3' basal subdomain from mutations in Dhr1 and its activator Utp14. We now incorporate the suggestion that mutations in Dhr1 and Utp14 slow Dhr1 to provide a longer window of opportunity for productive RNA folding, analogous to the effect of mutations in Prp28 or Prp5 that increase splicing efficiency on suboptimal substrates.

In figure 6 the authors make use of an auxin system to deplete Bud23 and to characterize the defect in ribosome biogenesis by sucrose density gradient sedimentation coupled to western and northern blotting. These data show a loss of Enp1 and Imp4 from the SSU processome position in the gradient, which does not agree with the overall conclusions that the authors make in Figures 1-5, which point to the stabilization of Imp4 binding to the SSU processome in the absence of Bud23. Instead the authors write that the retention of U3 at the SSU processome is surprising; however, retention of U3 is expected if binding of Bud23 leads to progression of the SSU processome to the pre-40S ribosome as is accumulation of Imp4 and Enp1, which is not observed and is the more surprising result. The authors then lead into Figure 7 by describing a pull down experiment using Enp1-TAP saying (line 447-449) "Enp1-TAP is an ideal bait for these experiments as it binds to the SSU processome, upstream of Bud23 binding, and is released downstream of Bud23", but the authors just demonstrated (6 lines above) that Enp1-TAP is lost from the SSU processome in the absence of Bud23. We suggest that the pull down experiment is more relevant and in agreement with the author's hypothesis, while the gradient experiment may suffer from complexes falling apart during centrifugation, perhaps because steps have occurred "out-of-order" in the absence of Bud23. This apparent contradiction at least needs to be acknowledged if not resolved. Additionally, western probing against other factors may add insight into the results of the experiments in figure 6C.

We respectfully disagree with the reviewer's interpretation here and regret if it was due to a lack of clarity of our presentation. In this revision, we have clarified that we propose Bud23 is required during the transition of the SSU Processome to pre-40S and not for initiating this transition. That Enp1 is lost from the 90S region of the gradient reflects that the arrest point is downstream of the SSU Processome. We have previously reported that catalytic Dhr1 mutants and cold-sensitive Dhr1 mutants also lead to accumulation of particles in a ~45S position and not 90S position due to an arrest in progression of the assembly pathway (Sardana et al , RNA 2014; Sardana et al, PLOS Bio, 2015;

Zhu et al, MCB, 2016). Because we are proposing that Bud23 works in conjunction with Dhr1 to promote assembly of the pre-40S, we expect both to arrest the pathway downstream of the 90S, as we observe. We maintain that Enp1 is an appropriate bait for this experiment.

Reviewer #1 (Minor Comments):

Minor comments:

The A1 cleavage defect of the bud23 deletion cells, which are sick, may be an indirect consequence of accumulating SSU processomes, rather than a direct effect of Bud23; this should possibility should be acknowledged.

We now acknowledge this possibility at the end of the Bms1 subsection of the results.

Line 23: "3' domain" should either be defined in the abstract or omitted.

We have omitted the use of "3' domain" from the abstract and author summary, as suggested by the reviewer.

Reviewer #2 (General assessment and major comments (Required)):

Elife_05-02-2020-eLife-55805

In this manuscript Johnson and co-workers are using yeast genetics, complemented by gradient and pulldown analyses (Northern, western blot and mass spectrometry) of assembly intermediates to decipher the role of the assembly factor Bud23 in 40S ribosome maturation.

The work represents the kind of careful, thoughtful and creative analysis that we have come to expect from the Johnson lab, and provides exciting new information about a network of proteins that promotes 40S assembly. The genetic interactions are interpreted in the context of the recent cryo-EM structures, which illuminates their role, and provides one of the first examples of how these structures could be used for any kind of mechanistic analysis.

In principle, this work is definitely of the impact and quality to be considered for publication in Elife. Nevertheless, I feel some revisions are appropriate. I also am not convinced that the current data actually strongly support the proposed model for the role of Bud23, Dhr1 and Utp14.

Additional detail is provided below.

Major points:

1. I would encourage the authors to provide one Figure that maps ALL the mutations and proteins in this manuscript at the same time. This could be at the beginning or the end of the manuscript.

We appreciate the intent of this comment. However, the SSU Processome is an extremely complex structure and we do not feel as though it is possible to generate a single figure that maps all mutations in a meaningful fashion. Figure 1 provides a contextual overview of the domain of the SSU Processome that we focus on. It also lists all factors in which we found mutations and shows their structures. Figure S3 extends this to include regions of Utp14 for which we have structure. But the region of Utp14 in which we identified mutations, and all of Dhr1 along with regions of Utp2 in which we also found mutations are not resolved in existing structures. We believe that mapping the individual mutations to structure in separate panels is the most effective way to present them. However, we have now included a list all the mutations in Supplemental File 1 to make it easier to consider all of the mutations at once.

2. The observation that disruption of the Utp14/Dhr1 interface suppresses Bud23 really is inconsistent with the model that Bud23 promotes unwinding by Utp14/Dhr1. The authors need to take these data into account in their proposed model. There is one possible model that could perhaps reconcile these observations. In this model the mutations stabilize the ADP-bound form of Dhr1, which may not bind Utp14...Minimally, the suggested model would predict that the Bud23 suppressors in here also suppress DHr1 mutants. Furthermore, the suggested models also predict that Bud23 deletion should be epistatic with Bms1 GTPase mutants. These two predictions can be easily tested using reagents the authors have in hand.

Please see our response to point 1 from reviewer 1. We have clarified that we do not propose Bud23 directly promotes Dhr1 unwinding but rather is needed for the proper structural context of the Dhr1 substrate to promote productive unwinding.

3. More generally, I do feel like a more mechanistic analysis of the effects from the suppressors would be in order for a manuscript in Elife. Currently, the authors demonstrate that the suppressors rescue the RNA processing defects. That is expected given the rescue in growth. The real question is how? Minimally, the authors should investigate AF recruitment and gradient as they have done in Figure 6 and S6 (for one suppressor). Also, if I were doing these experiments, I would compare the Enp1-TAP pulldown in Rcl1 depleted cells with and without Bud23. That way they are blocked prior to A2 no matter what, and one now looks truly at the differences between +/- Bud23; not the difference between 90S and 40S molecules.

We previously investigated the effects of bud23Δ-suppressing mutations in Utp14 and Utp2 by characterizing the sedimentation of AFs in sucrose gradients, subcellular localization of AFs, and by co-immunoprecipitation of preribosomes with AFs (Sardana et al, RNA, 2013). In that work, it was shown that deletion of BUD23 caused significant changes (i.e. loss of 20S rRNA association, nuclear mislocalizaton of SSU Processome factors, and changes in steady-state distribution through sucrose gradients). Suppressors of bud23Δ partially corrected these defects. Thus, we think that additional characterization of partially suppressed mutants would not be useful nor be new information.

We respectfully disagree with the suggestion to use Rcl1-depletion to understand Bud23 function. Depletion of Rcl1 slows A0, A1, and A2 processing (Billy et al, EMBO Journal, 2000), a phenotype common to factors needed for SSU Processome assembly (Osheim et al, Molecular Cell,

2004 & Dragon et al, Nature, 2002). As Bud23 is expected to act downstream of SSU Processome assembly and the A0 cleavage, we expect that depletion of Rcl1 would block the pathway upstream of Bud23 function and would not be informative.

Reviewer #2 (Minor Comments):

4. l.106: I do not think it is correct that A3 can be used instead of A2. I believe the data suggest that when A2 does not happen, A3 still continues to occur, resulting in 23S. However, in contrast to 20S, 23S cannot make 18S. This is of minor relevance for this work, but it should be clarified.

Reviewer #3 expressed similar sentiments. Please see our response to comments under Reviewer #3.

5. l.115: Bud23 is not present in the 90S structures. How certain are we of this? Is it possible that it is there, but disordered, like many other factors? I guess the structures of the human intermediate suggest that this is unlikely, but perhaps some additional clarification could be helpful.

We have changed the words "not present" to "not identified". We also now note in the main text that the Bud23 binding site is occupied by Emg1 in the SSU Processome, making it extremely unlikely that Bud23 is present in the complete SSU Processome.

6. L.207; there are a number of references regarding Imp3,Imp4,Mpp10 entry into 90S from the Baserga lab, as well as from Mercedes Dosil's lab.

Thank you for pointing this out; we now cite relevant work from the Baserga lab. We do not cite the work from the Dosil lab (Pérez-Fernández et al, NAR, 2011) because it does not place Imp4-Mpp10-Imp3 as entering the SSU Processome assembly pathway early which is inconsistent with other generally accepted lines of evidence that suggests it does (Chaker-Margot et al, NSMB, 2015; Zhang et al, Genes & Dev, 2016; Hunziker et al, eLife, 2019).

7. Ll. 480: The more likely interpretation of the "accumulation" of Rio1 in the Bud23 strain is that it is an artifact, due to the fact that it is really very minor in wt strains. Ie the change is big.

We believe that the presence of Rio1 in this particle is real as spectral counts and percent coverage were comparable to factors that are expected to be in the complex. However, this is a minor point and we do not base any conclusions or models on the observed presence of Rio1.

Reviewer #3 (General assessment and major comments (Required)):

The present paper continues a series of genetic analyses based on the observation that bud23Δ mutants generate spontaneous suppressors in other early-acting ribosome synthesis factors. The authors isolate and identify many new suppressor mutations in known targets, as well as in two genes not previously picked up in this screen. They then make good use of the published pre-ribosome structure data to interpret the mutations and design specific mutations to test specific,

predicted interactions. This allows them to make some interesting and plausible predictions on the role of Bud23 in helping dissociate factors from the 3' minor domain at the SSU processome => pre-40S transition. This is nice work but, as the authors point out, biochemistry and structural analyses will be needed to validate these hypotheses.

Fig. 6A and S5, S6 - provide clearest data in MS for requirement for Bud23 during SSU processome disassembly, but it is less clear how direct/active this is. It is evident from these and many previous analyses, that an intricate network of interactions is needed for correct assembly and timely disassembly of the processome. Loss of Bud23 only partially disrupts this structure, since the deletion strain is viable, but it seems likely that other partial loss of function mutations could be generated with similar phenotypes.

Overall, this is good work that I enjoyed reading. The experimental work is technically solid but almost all of the key conclusions are based on supposition. This does offer a number of potentially useful starting hypotheses for future work but, regrettably, I feel that the work lacks the significance for publication in eLife. It appears suited to a more specialist RNA journal, where I would be happy to recommend immediate publication.

Reviewer #3 (Minor Comments):

P27 "Cleavage at the A2 site appears to reflect co-transcriptional processing while cleavage at the A3 site is observed in post-transcriptional processing"
This is not really what has been reported. Cleavage at A2 and A3 always occurs, on both the "co-transcriptional" and "post-transcriptional" processing pathways. However, A3 is cleaved post-transcriptionally in both cases, whereas the timing of A2 (and A1) cleavage defines whether the pathway is termed co- vs post-transcriptional.

Thank you for bringing this to our attention; we have rephrased our language regarding A2 and A3 cleavage throughout the manuscript. We have also modified Fig S1 to correctly reflect the alternative processing pathways.
