# Supplementary material for: Bud23 promotes the final disassembly of the small subunit Processome in Saccharomyces cerevisiae
Source: PLoS Genet. 2020 Dec 11;16(12):e1009215. doi: 10.1371/journal.pgen.1009215 (PMC7758049; doi:10.1371/journal.pgen.1009215)
Supplement: Attachment — Submitted filename: Response to Reviewers.docx [file pgen.1009215.s014.docx]

Dear Arlen,

Thank you very much for submitting your Research Article entitled 'Bud23 promotes the progression of the Small Subunit Processome to the pre-40S ribosome in Saccharomyces cerevisiae' to PLOS Genetics. Your manuscript was fully evaluated at the editorial level and by three independent peer reviewers. The reviewers appreciated the attention to an important topic but identified some aspects of the manuscript that should be improved.

We therefore ask you to modify the manuscript according to the review recommendations before we can consider your manuscript for acceptance. Your revisions should address the specific points made by each reviewer, as summarized in my comments to you.

"The main thrust of your argument is that each of the mutations destabilizes the 90S intermediate. If so, one would expect that these mutations might have growth defects by themselves. For a subset of mutants this is shown. I am certain that you have these data, and think it would be useful to include them.

To more directly assess whether individual proteins are destabilized, it would be terrific if you could show that one or two of the mutant protein sediment outside of pre-ribosomes. as a proof that this is true...i dont think it would be necessary to do this with each mutant, or each protein....one or two proteins (whatever you have a good antibody for) in one or two mutants...(with wt bud23)."

… … …

Dear Arlen,

your manuscript has now been evaluated by three reviewers as well as by myself. I am assuming that the comments will be appended...

Two of the reviewers are very favorable, and they have seen previous iterations.

A third reviewer has a couple of extra comments, which I think are worth considering.

The main thrust of your argument is that each of the mutations destabilizes the 90S intermediate. If so, one would expect that these mutations might have growth defects by themselves. For a subset of mutants this is shown. I am certain that you have these data, and think it would be useful to include them. To more directly assess whether individual proteins are destabilized, it would be terrific if you could show that one or two of the mutant protein sediment outside of pre-ribosomes. as a proof that this is true...i dont think it would be necessary to do this with each mutant, or each protein....one or two proteins (whatever you have a good antibody for) in one or two mutants...(with wt bud23)...

Stay safe!

Best,

Katrin

We thank you, Katrin, and all of the reviewers for your time and helpful commentary. We have considered each of the reviewers’ points individually and detail our responses below. One of the major concerns was that our argument that the suppressing mutations destabilize protein-protein and protein-RNA interfaces within the SSU Processome and such mutations should cause growth defects and lead to increased levels of free proteins. To address this, we now include supplementary data showing that, in fact, single-point mutants of *IMP4*, *DHR1*, and *BMS1* showed no obvious growth defects in *BUD23*-replete cells (Figure S4, S5 and S7). We also show that combining mutations to enhance their defect on protein function can lead to growth defects and even lethality. However, such mutants do not result in free protein, presumably because of the extensive interaction network within the SSU Processome. We hope that our manuscript is now acceptable for publication in *PLOS Genetics*.

Reviewer's Responses to Questions

Comments to the Authors:

Please note here if the review is uploaded as an attachment.

Reviewer #1: Here Black et al. have devised a series of genetic and biochemical studies to elucidate the role of Bud23 in the disassembly of the small subunit processome. By screening for suppressors of a Bud23 deletion, the authors have identified a number of suppressor mutations in a set of assembly factors that functionally connect the 3’ domain with the unwinding of the U3-18S RNA duplexes. The authors propose a model in which the binding of Bud23 to pre-rRNA aids in the disassembly of additional ribosome assembly factors from the SSU processome. This model also postulates that in the absence of Bud23, mutations that weaken protein-protein interactions or reduce productive enzymatic activities (GTPase or RNA helicase activities by Bms1 and Dhr1 respectively) can compensate for an otherwise suboptimal compaction of the SSU processome into a pre-40S particle. This in-depth study provides important inroads for subsequent structural and mechanistic studies of the SSU processome and is of general interest for researchers studying RNA-protein interactions and larger assemblies.

It is the opinion of this reviewer, that the authors have addressed all comments that were raised during a previous round of review and that provided that the minor comment listed below is addressed, this manuscript should be published in PLOS Genetics.

Minor comment:

In figure 6C the authors show that in response to the depletion of Bud23, the assembly factor Enp1 is largely shifted to lower molecular weight species while Imp4 is still present at a reduced level in higher molecular weight complexes. This would suggest that there are different sets of complexes resolved on the sucrose gradient that are currently not identified. In support of this, the purified RNPs containing Enp1 (Fig. 7A) in the presence or absence of Bud23 further indicate that several additional ribosome assembly factors (including Kre33, Utp2 and Bfr2 among others) are enriched upon depletion of Bud23. Figure 6C therefore only provides an incomplete picture of the composition and complexity of the mixture of particles that are contained within the Bud23 depleted sample shown in Fig.7A.

To address this, the authors should provide Western blots for enriched proteins (Kre33, Utp2, Bfr2) or reduced proteins (Tsr1 or Rio2) for figure 6c.

We thank the reviewer for their positive review. We agree that there is likely a spectrum of discrete particles that accumulate in the absence of Bud23. We have added some language to the main text that address this possibility (lines 530-531).

Reviewer #2: I have previously reviewed this paper for another journal and my specific comments have been suitably addressed.

The present paper continues a series of genetic analyses based on the observation that bud23∆ mutants generate spontaneous suppressors in other early-acting ribosome synthesis factors. The authors isolate and identify many new suppressor mutations in known targets, as well as in two genes not previously picked up in this screen. They then make good use of the published pre-ribosome structure data to interpret the mutations and design specific mutations to test specific, predicted interactions. This allows them to make some interesting and plausible predictions on the role of Bud23 in helping dissociate factors from the 3' minor domain at the SSU processome => pre-40S transition. These are good genetic analyses although, as the authors point out, biochemistry and structural analyses will be needed to validate the hypotheses.

Figs. 7, S5, S6 - provide clearest data in MS for requirement for Bud23 during SSU processome disassembly. It is less clear how direct/active is this function, but the data support the existence of an intricate network of interactions needed for correct assembly and timely disassembly of the processome. These are nicely presented in Fig. 8.

The experimental work is technically solid and the conclusions offer several significant starting hypotheses for future work. Overall, the work appears well suited to publication in PLoS Gen.

We thank the reviewer for their time and careful reading of our manuscript and their complimentary comments.

Reviewer #3: In this manuscript the Johnson laboratory nicely takes benefit of recent structural achievements in the ribosome biogenesis field to better rationalize the structural/functional basis of extra-genic suppressors of bud23 deletion, a non-essential trans-acting factor involved in SSU biogenesis.

Based on this and additional analyses, the authors proposed a model where Bud23 promotes disruption of rRNA/protein and protein/protein interactions network, thereby leading to the release of several ribosome biogenesis contributing to this network and enabling SSU progression/transition to later steps of SSU assembly.

Whereas I find the observation very interesting, I am concerned about the lack of sufficient biochemical analysis convincingly supporting the major implication of the authors interpretation. I feel that the authors should provide more compelling mechanistic insights going beyond their valid, but not validated, hypothesis.

Specific comments:

1) The main conclusion of this study, is that the bud23 extra-genic suppressor mutations may individually decrease the interaction properties of the protein/protein and protein/rRNA interaction network, enabling the “collective” release of ribosome biogenesis factors contributing to this interaction network.

One implication is that independently of the nature of the mutation and affected factors, each individual suppressing mutation event is sufficient to modify the biochemical property of the full interaction network as such that it can bypass Bud23 function. In other words, whereas diverse, the suppressor mutations are functionally equivalent. It is interesting, as it is conceptually very different than a cooperative effect due to suppressor mutations distributed across an interaction network that would sum-up and collectively facilitate progression of SSU maturation in the absence of Bud23.

However, the experimental basis to better understand this intriguing observation and validate the postulated hypothesis is not fully convincing.

Additional efforts should be made to directly substantiate the decrease interactions of exemplary mutated ribosome biogenesis factors and members of the interaction network from purified pre-ribosomal subunits in a bud23 null background. For example, using the implicated factors as bait would clarify their direct and respective behaviour along the maturation pathway. Similarly, what are the consequences of introducing exemplary individual suppressor mutation in a Bud23 wildtype background? Are there any biochemical consequences on the interaction behaviour of this interaction network with pre-ribosomal particles? I would somehow expect a similar biochemical perturbation on the proposed interaction network. What happens to Bud23 in this case, does it get partially “excluded” from the maturation process?

We investigated the effects that the single point mutations of *IMP4*, *BMS1*, and *DHR1* had on growth in *BUD23*-replete cells and found that they appeared to complement as well as the wild-type alleles of these genes. We have added these additional results as new Figures S4A, S5,& S7. To the reviewer’s latter point, we determined if mutants would lose interaction with ribosomal precursors. For this, we focused on the interaction between Imp4 and the 3’ basal subdomain rRNA; however, since the single-point mutants showed no obvious growth defects we combined several mutations in an attempt to generate mutants with measurable defects. We generated double mutants and a quadruple Imp4 mutant (Imp4-TSMD) which contained the amino acid substitutions S93T, R94S, R116M, and N118D. Indeed, this mutant was inviable and was surprisingly strongly dominant negative in both *BUD23*-replete and *bud23*∆ cells (Figure S4A & B). We then ectopically expressed Imp4 wild-type or Imp4-TSMD C-terminally tagged with 13xMYC and monitored their association with pre-ribosomes by sucrose density gradient (Figure S4C). From this we saw that both wild-type and mutant proteins behaved similarly and sedimented throughout the gradient suggesting their association with pre-ribosomal complexes. Like many SSU Processome factors, Imp4 forms contacts with multiple assembly factors and rRNA elements. Thus, while it’s reasonable to think that these mutants would lose association with the SSU Processome, we conclude that the extensive network of protein-protein and protein-RNA interactions within the processome maintain association of the mutant proteins. Indeed, the dominant negative effect of the Imp4-TSMD mutant protein must be due to its continued association with binding partners. We discuss these results at length in the main text at lines 250-276.

2) Unless, I got this wrong, the experimental design presented in Figure 5 is not very helpful. Considering the bud23 suppressors are individual/unique events (see also above), why testing combination of multiple mutations found in Dhr1 for these interaction analyses? Are the single mutations not sufficient to modify the Dhr1/Utp14 binding property?

See our response to question 2 above. In addition, we previously reported Utp14 single point mutants that suppress the defects of *bud23*∆ cells (Zhu et al MCB 2016). All of these amino acid substitutions localized to a loop of Utp14 needed for its efficient interaction with and activation of Dhr1. On their own, single substitutions were unable to disrupt the interaction with Dhr1 assayed by yeast two-hybrid analysis. The loss-of-interaction phenotype could only be seen when multiple substitutions were combined. In this case, we could also measure reduced activation when multiple mutations were combined. Thus, here we reasoned that we would need to combine multiple substitutions within Dhr1 in order to observe any impact that they could have on the Dhr1-Utp14 interaction. As a result, we did not assay whether or not *dhr1* mutants harboring single amino acid substitutions. We have added some language to the text to more clearly explain our reasoning for making such combinatorial mutants of Dhr1.
