# Supplementary material for: Bud23 promotes the final disassembly of the small subunit Processome in Saccharomyces cerevisiae
Source: PLoS Genet. 2020 Dec 11;16(12):e1009215. doi: 10.1371/journal.pgen.1009215 (PMC7758049; doi:10.1371/journal.pgen.1009215)
Supplement: Attachment — Submitted filename: Response to Reviewers Comments Revision 2.docx [file pgen.1009215.s015.docx]

Response to Reviewer’s and editor’s comments

Nonetheless, I also suspect that the impact of the work would benefit from adding the information from these structures into the manuscript (and potentially revising details of your model). I want to very strongly urge you to do this because i think it will greatly increase the longevity of this study, and its impact. Naturally, this does not require any more experimentation, and I suspect you have already done this now that the pdb coordinates are released.

Best,

Katrin

*We thank you for the opportunity to revise aspects of our manuscript in light of the new structures of disassembling SSU Processomes from the Beckmann group. We have made various small changes throughout the text for clarity, however, we have extensively revised the Discussion. (Changes are tracked in the text.) We suspect that the final complex, “Dis-C”, that the Beckmann group reports is very close in nature to the particle to which Bud23 is recruited. Notably, all of the factors in which we find bud23∆ bypassing mutations are retained on this structure. Previously we had argued that Bud23 is promoting rearrangements that promote collapse of the Processome rRNA into a compact pre-40S. We can now be more specific and propose that Bud23 promotes the collapse of the 3’ major domain in head formation to finalize the transition of the SSU Processome to pre-40S. The completely revised model is presented in a new Fig 8.*

Reviewer #3: I sincerely appreciate the efforts made by the authors to provide additional results and to take the comments/suggestions into consideration, however, the functional and biochemical analysis, as analysed so far, do not provide the necessary compelling and logical evidences supporting the interpretation and model proposed by the authors of how Bud23 may fulfil its function.

There is no doubt that the data are of high standard and in case I have misunderstood the authors’ interpretations and their biological implications, I hope they will make sufficient efforts to alleviate the possible source(s) of this misunderstanding, if any.

As indicated in my previous comments, there are significant logical gap and inconsistencies which still needs to be fully addressed or the proposed model needs to be substantially revised to reflect the available data. Based on the current experimental evidence the proposed model is not very intuitive and the arguments are not fully convincing (at least to me).

In the case that the authors have not fully understood my previous comments/suggestions and to alleviate any ambiguity, I have reformulated my main concerns below in order to complement and clarify, if necessary, my previous comments.

The main logical problem in the authors argumentation is to reconcile the fact that individual point mutations in different assembly factor/r-protein bypass the bud23 growth-defect and presumably its function independently of each other’s. In fact, based on the nature and structural environment of the observed extra-genic suppressors, the authors interpret their genetic interactions study in a more general physical-interaction network and accordingly conclude that Bud23 influence the disassembly/stability of this physical (genetic) network. The implication of this interpretation is that the suppressor mutations individually contribute to biochemical perturbation of this physical interaction network.

Considering that the genetic suppression is based on single and individual mutation events across various factors. This implies that any of these should have some common/similar functional effects/consequences.

*Indeed, we do show that all bypassing mutants that we tested here and in previous publications, representing mutations in all the factors identified, all have a common output of partially restoring A2 cleavage, 40S production and cellular growth. See Figures 2A, 2E, 2F, 3A, 3B, 4A, 4B, and 4C of this manuscript and our previous publications for the Utp14 mutants (Zhu et al, MCB, 2016), the Dhr1 mutants (Sardana et al, MCB, 2014), and the Utp2/Utp14 mutants (Sardana et al, RNA, 2013).*

According to the authors: destabilization of protein/protein interaction protein/RNA interactions. Among the examplary anaylysis that would/could support this interpretation, the combination of multiple “suppressor mutations” are necessary to observe the expected functional consequences in agreement with the author’s model (e.g. Dhr1-Utp14 interactions). However, and in contracdiction to the proposed model, when isolated, the individual mutations have no apparent biochemical consequences (e.g. Dhr1-Utp14 interactions/rebuttal).

*We do not agree with this reviewer’s conclusion that our results present a contradiction. In our previous in vitro work with mutations in Utp14 that inhibit activation of Dhr1, we showed that combining multiple mutations had a measurable impact on Dhr1 activation and this was enhanced when the multiple suppressing mutations were all alanine substitutions (Zhu et al, MCB, 2016). Deletion of the region had an even more profound defect. The in vitro results correlated with their impact on cell fitness. Thus, there was a correlation between the severity of the amino acid sequence disruption, the biochemical readout and cell fitness.*

Strikingly, combining suppressor mutations, when tested, are not able to suppress the Bud23 loss of function anymore and do not show obvious differences in their biochemical behaviour, when compare to WT (e.g. Imp4 analysis Supp Fig4).

An additional (new) argumentation line or alternative (?) explanation is that the extensive interaction network within the 90S would somehow sustain the interaction to “normal” level or the changes might be too subtle to be measured using “standard conditions”. This could be true, but this remains a very vague justification and rather contradicting with the advertised authors interpretation. Challenging the interactions strength of exemplary individual suppressor mutations and/or the bud23-network members to pre-ribosome using more stringent biochemical conditions (e.g. increasing salt concentrations) could help to validate this possibility.

*Mutations in essential factors that bypass the loss of Bud23∆ must be in a fine balance between perturbing function sufficiently to bypass the loss of Bud23 function without significantly perturbing the essential function of the essential factor. It is easy to imagine that greater disruption of an essential function or interaction will be deleterious or even lethal. In our last revision, we demonstrated that single point mutations in Imp4 that bypass bud23∆ have no obvious defect on their own whereas combining these into double and quadruple mutants leads to reduced or loss of bud23∆ suppression and reduced or loss of function, respectively. That single point mutations in Imp4 do not significantly disrupt the SSU Processome function is understandable when you consider the number of contacts that Imp4 makes. Imp4 interacts with the pre-rRNA, 5’-ETS, U3, Utp1, Bms1, Rps16, Faf1, Mpp10, and Imp3. Thus, any single point mutation is well buffered by its extensive interaction network and allows it to maintain an interaction with the SSU Processome complex.*

*We suspect that because of the reduction in complexity of the particle as it matures from an SSU Processome to state Dis-C, the buffering effect of contacts is reduced as the number of contacts is reduced. This may allow point mutations in individual factors to have a greater effect on the Dis-C complex, which we propose is close to the structure that Bud23 acts on, than on the SSU Processome. This may, in part, explain how these mutations can bypass bud23∆ without significantly perturbing cell growth. We did not discuss this concept in the manuscript as we felt that it is too nuanced and speculative.*

*None of the factors that we have identified mutations in are involved in simple binary interactions in vivo where one might expect to observe differential stability by modifying buffer conditions to disrupt ionic or hydrophobic interactions, as this reviewer suggests. Indeed, we have done a considerable amount of work along these lines with Imp4 and concluded that it’s interaction with the Processome is not easily disrupted, even in the case of a lethal quadruple mutation, as shown in the supplemental data.*

*We do, however, show that single amino acid substitutions in Utp2 or Imp4 at the Utp2-Imp4 interface reduce their interaction, assayed using yeast 2 hybrid (Fig. 3D and 3F). We don’t know if the yeast 2 hybrid assay reduces this to, essentially, a binary interaction but it likely simplifies the system making it easier to detect a loss of interaction. We believe our results are all self-consistent and easily interpreted both in terms of genetic and physical interactions.*

*We hope that our revisions to accommodate the new structures from the Beckmann group along with our further explanations to address the concerns of reviewer 3, which we think are based on incorrect interpretation of genetic interactions, have adequately addressed your concerns and that you will now find this manuscript acceptable for publication.*
